# Malaria crystalloids: specialized structures for parasite transmission?

**DOI:** 10.1016/j.pt.2010.12.004

**Published:** 2011-03

**Authors:** Johannes T. Dessens, Sadia Saeed, Annie Z. Tremp, Victoria Carter

**Affiliations:** Department of Pathogen Molecular Biology, London School of Hygiene & Tropical Medicine, Keppel Street, London WC1E 7HT, United Kingdom

## Abstract

Malaria parasites possess many unique subcellular structures and organelles that are essential for the successful completion of the complex life cycle of *Plasmodium* in the vertebrate host and mosquito vector. Among these are the crystalloids: transient structures whose presence is restricted to the mosquito-specific ookinete and young oocyst stages of the parasite. Nearly five decades after they were first described, the crystalloids are back in the spotlight, with recent discoveries pointing to an important role in protein trafficking and sporozoite transmission that could be exploited as new targets for control of malaria transmission.

## Crystalloids and malaria transmission

Malaria remains a devastating parasitic infection in humans, causing an estimated 0.5 billion clinical cases annually and more than one million deaths [1]. Malaria control is hampered by widespread resistance to antimalarial chemotherapy and insecticides, emphasizing the urgent need for novel intervention strategies. Control of parasite transmission by mosquitoes is considered a vital component of a successful malaria-control programme.

Malaria transmission begins with the uptake of gametocytes in the blood meal of a feeding vector mosquito, which initiates gametogenesis and fertilization. Zygotes transform over a 16–20 hour period into ookinetes. These cross the midgut epithelium and then transform into oocysts. In the following two to three weeks, oocysts grow and generate thousands of progeny sporozoites. The sporozoites invade and inhabit the salivary glands of the insect, where they await transmission to a new host upon mosquito bite to initiate new malaria infections.

Crystalloids are unique subcellular structures of malaria parasites that are implicated in malaria transmission by nature of their exclusive presence in ookinetes and young oocysts. As early as 1969 it was postulated that the crystalloids could constitute a reservoir of protein synthesized in the gametocyte that is used during oocyst development [2], but experimental evidence regarding the origins and functions of the crystalloids has remained elusive. Recent studies, however, have demonstrated a functional link between the crystalloids and a family of *Plasmodium* LCCL proteins (see below) that are essential for sporozoite transmission. This discovery has raised new interest in this intriguing parasite structure from a cell biological perspective and as a potential target for control of malaria transmission.

In this article, a new interpretation of the available literature on crystalloids is entertained. That is, the crystalloids could be composite vesicular organelles designed to traffic molecules from the gametocyte to the young oocyst, where the crystalloid subunits dissociate and enter a vesicular pathway to deliver their cargo.

## What are malaria crystalloids?

**Figure 1:**
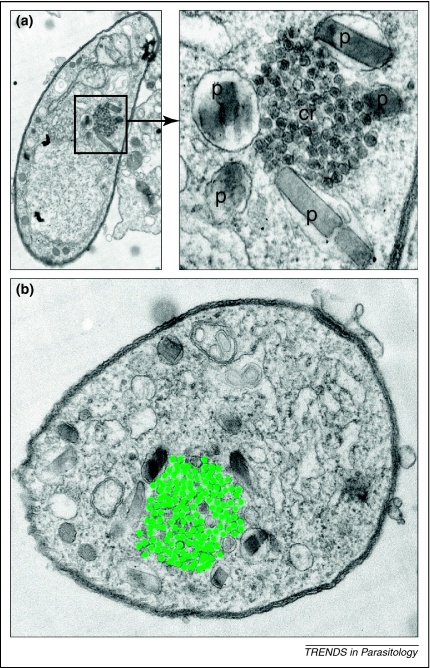
Ultrastructure of the malaria crystalloid in *Plasmodium berghei* ookinetes. **(a)** Transmission EM image of a whole ookinete section. A subsection (boxed) is shown at higher magnification in the right-hand panel that more clearly shows the crystalloid (cr) surrounded by vacuoles containing malaria pigment (p). **(b)** TEM image of an ookinete cross-section. The crystalloid subunit particles have been highlighted in green. In part reproduced from [10].

In the context of malaria, the term ‘crystalloids’ was first used by Garnham and colleagues in 1962 to describe the ‘unusual cytoplasmic structures’ they observed by electron microscopy (EM) in the ookinetes of avian and simian *Plasmodium* species [3]. Similar structures were later discovered in the ookinetes of murine and human *Plasmodium* species [2,4], indicating that they are conserved structures throughout the genus. Crystalloids appear under EM as cytoplasmic aggregations of small irregular spherical particles of diameter 25 to 40 nm (Figure 1). The number of crystalloids per ookinete varies between species, but is typically one to three, whereas crystalloid size varies in diameter between 0.5 μm and 2.0 μm [5]. In the ookinetes of *Plasmodium berghei*, two crystalloids are typically present that are surrounded by multiple vacuoles containing hemozoin (a byproduct of haemoglobin digestion in the food vacuoles) (Figure 1) [2,6]. The crystalloids are carried over into the downstream life stage during ookinete-to-oocyst transformation, but are typically absent from older oocysts [2]. Depending on the *Plasmodium* species (and conceivably also preparation of the EM specimen), the organisation of the subunit particles in the crystalloids tends to be geometrical. This gives the appearance of a crystal-like structure, which presumably gave rise to the name ‘crystalloid’. These geometrically configured crystalloids resemble viral inclusions, which led some authors to believe that the origin of the crystalloid was the result of a viral infection of the parasite [7,8]. The transient nature of the crystalloids led other researchers at the time to postulate that the crystalloids provided a reservoir of protein [2] or a lipoprotein energy store [9] that is necessary during oocyst development.

**Figure 2:**
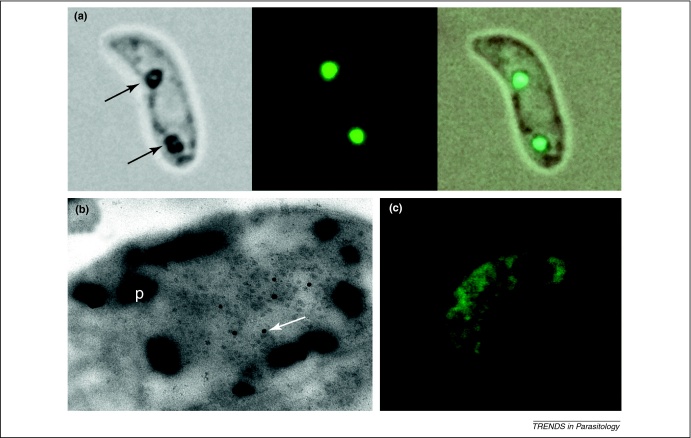
*Pb*SR is associated with crystalloids. **(a)** Image of a *P. berghei* ookinete expressing GFP-tagged *Pb*SR showing co-localization of the fluorescent spots with malaria pigment (arrows). **(b)** Immunogold labelling (arrow) of the crystalloid with anti-GFP antibodies on ookinetes expressing GFP-tagged *Pb*SR. This image clearly shows how the crystalloid is surrounded by pigment (p). **(c)** Typical localization of GFP-tagged dysfunctional *Pb*SR in an ookinete showing the absence of the crystalloid-associated spots. In part reproduced from [10].

A breakthrough in our understanding of crystalloids came recently when the first parasite protein to be directly involved with crystalloids was discovered in *P. berghei*, thereby discarding the hypothesis of a viral origin [10]. The protein in question, *Pb*SR, is a member of the *Plasmodium* LCCL protein family and had previously been shown by targeted gene disruption to be important for sporozoite development in the oocyst [11]. Using a series of genetically modified parasite lines, it was shown by confocal microscopy that fluorescent protein-tagged versions of *Pb*SR generated two fluorescent spots associated with the malaria pigment in *P. berghei* ookinetes (Figure 2(a)) [10]. These spots were shown by immuno-EM to correspond to crystalloids (Figure 2(b)). Parasites expressing similarly tagged but dysfunctional mutant versions of *Pb*SR did not form these spots in ookinetes (Figure 2(c)), and the study went on to show by EM that such ookinetes were devoid of crystalloids [10]. The same was found for parasite lines lacking *Pb*SR expression [10]. These findings not only established a functional link between *Pb*SR expression and crystalloid formation, but provide strong support for a central role of the crystalloids in sporozoite development in the oocyst and, hence, in malaria transmission [10].

## The *Plasmodium* LCCL protein family

**Figure 3:**
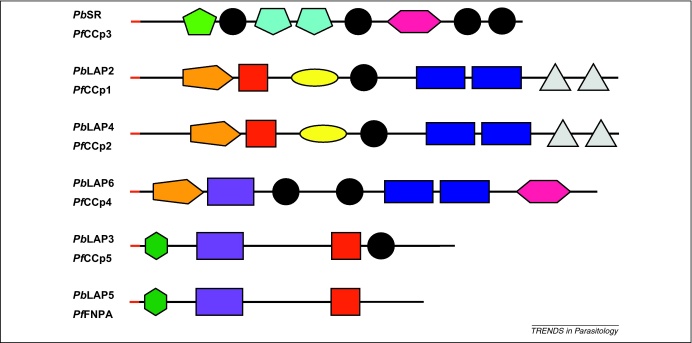
The *Plasmodium* LCCL protein family (schematic). The names given are the most commonly used for these proteins in *P. berghei* (*Pb*) and *P. falciparum* (*Pf*). All proteins possess a predicted amino-terminal endoplasmic reticulum signal peptide (red). Various domains are shown with significant homologies to known protein domains. See Pfam/Smart entries (in brackets) for further detailed information on these domains. Black: domain related to *Limulus* coagulation factor C, Coch-5b2 and Lgl1 (LCCL) domain (Pfam03815, Smart00603); light green: domain related to polycystin-1, lipoxygenase, alpha-toxin (PLAT) domain (Pfam01477, Smart00308); light-blue: domain related to scavenger receptor cysteine-rich (SRCR) domain (Pfam00530, Smart00202); pink: domain related to pentaxin/laminin-G domain (Pfam00354, Smart00159); orange: domain related to ricin-type beta trefoil lectin domain (Pfam00161, Smart00458); red: domain related to coagulation factor 5/8 carboxy-terminal domain/discoidin domain (Pfam00754, Smart00231); yellow: domain related to fibrillar collagen (COLFI) carboxy-terminal domain (Pfam01410, Smart00038), also known as neurexin and collagen-related (NEC) domain; dark-blue: Levanase-like domain; purple: domain related to anthrax protective antigen domain (Pfam07691); dark green: domain related to fibronectin type-II domain (Pfam00040, Smart00059); grey: domains related to apicomplexan-specific cysteine-rich domain. This diagram was compiled from [12–15].

The *Plasmodium* LCCL protein family is a group of six highly conserved and structurally related proteins with a modular architecture consisting of multiple domains implicated in the binding of proteins, lipids and carbohydrates [11–15] (Figure 3). The family members are typified by, and named after, the *Limulus* clotting factor C, Coch-5b2, Lgl1 (LCCL) domain [16] that is present in single or multiple copies in all but one family member (Figure 3). LCCL proteins also possess a predicted endoplasmic reticulum (ER) signal peptide at the amino-terminus and lack other known organelle-targeting sequences which, combined with the presence of multiple adhesive-type domains, points to an extracellular function for these proteins [11,14]. Over the years, LCCL protein family members have been ascribed different names, including *Plasmodium* LCCL domain-containing proteins (*P*CCp), used mostly for *P. falciparum* [14], and *Plasmodium* LCCL-lectin adhesive-like proteins (*P*LAP), chiefly used for *P. berghei* [15]. Within the family, *P*LAP2 (*P*CCp1) and *P*LAP4 (*P*CCp2) are close structural paralogues with identical domain compositions and topologies (Figure 3) and are probably derived from a gene-duplication event [12,14]. The very similar domain architecture of *P*LAP3 (*P*CCp5) and *P*LAP5 (*P*FNPA) (Figure 3) also points to a vertical relationship (even though the LCCL domain is absent from the latter). Thus, it can be argued that LCCL proteins should be considered a family of only four structurally distinct members, two of which have duplicated gene copies.

The *P. berghei* scavenger receptor-like protein *Pb*SR was the first LCCL protein family member to be characterized by targeted gene disruption, and this study revealed an essential role in sporozoite development in the oocyst [11]. Accordingly, *Pb*SR knockout parasites readily form oocysts in vector mosquitoes that at first appear normal, but later fail to produce sporozoites. Interestingly, these oocysts reach a significantly larger final size than their sporulating counterparts [11]. It was also reported that the non-sporozoite-producing oocysts of *Pb*SR knockout parasites undergo substantial nuclear multiplication, indicating that the absence of *Pb*SR impacts on cytokinesis rather than nuclear division [11]. A subsequent study showed that *Pb*SR null mutants can give rise to a low level of sporulating oocysts in mosquitoes [10]. However, whereas the resulting sporozoites appeared normal with regards to their morphology, circumsporozoite protein expression on the surface, and gliding motility, they were not found in salivary glands, indicating that they are non-infective [10]. Very similar loss-of-function phenotypes have been reported in *P. berghei* for *Pb*LAP2, *Pb*LAP4, *Pb*LAP5 and *Pb*LAP6 [17,18]. In *P. falciparum*, disruption of *pfccp3* and, independently, *pfccp2*, showed no apparent adverse effect on sporozoite development in the oocyst, but again the resulting sporozoites did not reach the mosquito salivary glands [14]. Thus, *Plasmodium* LCCL proteins appear to play vital parts in the development, infectivity and transmission of sporozoites.

## Expression, localization and molecular interactions of *Plasmodium* LCCL proteins

*Pb*SR was initially reported to be expressed in sporozoites using indirect immunofluorescence [11,15]. However, a later GFP reporter study indicated that *Pb*SR expression was gametocyte-specific [19]. To further investigate this matter, genetically modified *P. berghei* parasite lines were generated that stably expressed versions of *Pb*SR tagged with red and green fluorescent protein (GFP). These studies clearly showed that *Pb*SR is expressed predominantly in female gametocytes and not in sporozoites [10]. These parasite lines displayed normal development throughout the life cycle, demonstrating that the protein tagging of *Pb*SR had not affected its function [10]. More recently, GFP-tagging experiments also demonstrated the gametocyte-specific expression of *Pb*LAP2 and *Pb*LAP3 [20], whereas an independent GFP reporter study also points to the gametocyte-specific expression of *Pb*LAP4 and *Pb*LAP6 [21]. Thus, there is now a clear consensus that all *P. berghei* LCCL protein family members are synthesized in gametocytes. This is in full agreement with the reported expression of the LCCL protein family members in *P. falciparum* gametocytes [14,22–25].

Using indirect immunofluorescence, *Pf*CCp molecules in *P. falciparum* gametocytes were found to be associated with the parasite plasma membrane and parasitophorous vacuole, indicating that these proteins may be secreted during gametogenesis [14,22–24]. *Pf*CCP1 has also been detected in *P. falciparum* culture medium [26], indicating that it could be secreted at an earlier stage. In gametocytes of *P. berghei*, the evidence for secretion of the LCCL proteins is not clear, although the observed pattern of distribution of *Pb*SR, *Pb*LAP2 and *Pb*LAP3 is reminiscent of a vesicular localization that could point to their secretion after gametogenesis [10,20]. There is, however, clear evidence in *P. berghei* that *Pb*SR, *Pb*LAP2 and *Pb*LAP3 are translocated to the ookinete crystalloids after fertilization, via which they are trafficked to the oocyst [10,20]. The fact that this has been observed for three structurally distinct LCCL proteins (and probably holds true also for the structural paralogues *Pb*LAP4 and *Pb*LAP5) suggests that crystalloid trafficking is intrinsically linked to the *Plasmodium* LCCL protein family [20]. This offers a plausible explanation for the considerable gap observed between their expression in the gametocyte and their apparent function in the oocyst, which comprises several days and developmental transitions. Accordingly, the crystalloids could constitute a component of an unusual protein trafficking mechanism designed to deliver proteins such as *Pb*SR from their site of synthesis (the gametocyte) to their site of action (the oocyst) [10,20]. The relative amounts of *Pb*SR found in gametocytes and ookinetes supports the notion that the female gametocyte is indeed the prime site of *Pb*SR synthesis, and that the protein is carried over into the downstream life stages [10]. This scenario is further supported by data showing that the *Pb*LAP proteins are inherited from the female parent, indicating that their expression is essential before fertilization [17].

Recently, mutant *P. berghei* parasite lines lacking expression of two *Pb*LAP family members at the same time were generated and analysed: *Pb*LAP1 and *Pb*LAP2, or *Pb*LAP2 and *Pb*LAP6 [21]. These double mutants displayed essentially the same phenotype as the single knockouts, indicating that there is no added adverse effect of multiple LCCL protein knockouts and hence that there is no functional redundancy between these family members [21]. The similar phenotypes of single or multiple LCCL protein knockout mutants, combined with the similar temporal and spatial expression patterns of the LCCL protein family members, is consistent with a scenario whereby these proteins operate in concert and possibly as a protein complex. This could be reflected by the observed formation of high-molecular-weight protein complexes of *Pb*SR observed in *P. berghei* gametocytes [10]. Indeed, extensive molecular interactions between different LCCL protein family members have recently been shown in co-immunoprecipitation experiments using gametocytes of *P. falciparum* [25]. Evidence has also been reported that *P. falciparum* LCCL proteins are co-dependently expressed at the protein level, further supporting a scenario of molecular interaction and shared molecular function of these molecules [23,25].

## Crystalloid formation and loss

Crystalloids can be found closely associated with budding sheets of smooth ER and Golgi-like vesicles, indicating that crystalloid formation/assembly occurs via these subcellular structures [2,6,27,28]. This is consistent with the predicted ER signal peptide present in all the LCCL proteins, and the reported formation of disulphide bonds of *Pb*SR in gametocytes [10]. The literature is conflicting as to whether the crystalloid subunits possess a limiting membrane, which is probably caused by differences in the preparation and analysis of EM specimens. Nonetheless, some of the higher-resolution EM images published clearly show a trilaminar unit membrane/phospholipid bilayer surrounding the individual subunits [4,29]. This is probably evidence that the crystalloid subunits are indeed membrane-bound and implies that the malaria crystalloid is a large cluster of small vesicles rather than a cytoplasmic particulate inclusion body. In oocysts, the subunit clustering of crystalloids has been described as being less organized, and crystalloid subunits are often observed dispersed in the cytoplasm [4,29]. Moreover, in older oocysts without intact crystalloids, subunit-like particles were observed within and close to the oocyst wall in *P. berghei*, *P. gallinaceum* and *P. cynomolgi* [29]. These combined observations suggest that the loss of the crystalloids in the growing oocyst results from the dissociation of its subunits, which then enter a vesicular pathway to deliver their cargo, possibly to the extracellular environment.

## Crystalloids and transmission intervention

The unusual composite architectures of the LCCL protein family members (Figure 3), combined with the high level of structural conservation between *Plasmodium* orthologues and the absence of these proteins from organisms outside the Apicomplexa, strongly argue for a conserved role of these molecules in *Plasmodium* biology. The link established between crystalloid formation, LCCL protein expression and sporozoite development has provided important new insights into the roles of crystalloids and LCCL proteins. This functional link also forms the basis for a potential new concept in control of parasite transmission. That is, sporozoite development can be targeted by interfering with proteins that are not themselves expressed in sporozoites, but instead are synthesized much earlier in the life cycle. In other words, if we could inhibit the function of molecules such as *Pb*SR that are involved in crystalloid formation, we could interfere indirectly with the development, infectivity and transmission of sporozoites. The fact that the LCCL proteins are already synthesized in blood-stage gametocytes further raises the attractive prospect that such processes could be targeted before the parasite enters the mosquito. This transmission-blocking strategy would therefore not rely on the uptake of drugs or antibodies by mosquitoes during blood feeding, and would complement existing strategies to reduce malaria transmission from human reservoirs [30]. There remain, however, many unanswered questions regarding the precise function and mode of action of the crystalloids and their associated molecules. Answering these questions could be the key to developing a new generation of measures to control malaria transmission.
